# Tropical cyclone exposure and risk of adverse birth outcomes in urban and rural areas of Georgia

**DOI:** 10.1088/2515-7620/ae718b

**Published:** 2026-06-02

**Authors:** Xuejuan Ning, Kai Chen, Zeyan Liew, Joshua L Warren, Seulkee Heo, Michelle L Bell, Nicole C Deziel

**Affiliations:** 1 Department of Environmental Health Sciences, Yale School of Public Health, New Haven, CT, United States of America; 2 Yale Center for Perinatal, Pediatric and Environmental Epidemiology, Yale School of Public Health, New Haven, CT, United States of America; 3 Department of Biostatistics, Yale School of Public Health, New Haven, CT, United States of America; 4 Yale School of Environment, New Haven, CT, United States of America

**Keywords:** natural disasters, climate, birth outcomes, environmental epidemiology, environmental disparities

## Abstract

Tropical cyclones (TCs) are highly destructive weather disasters. Prior studies of TCs and birth outcomes often examined single storms or single TC characteristics (e.g. maximum wind speed) and rarely assessed urban/rural differences. To better understand TC impacts on perinatal health, we evaluated associations between multiple TC exposure metrics and several adverse birth outcomes and considered urban–rural variations. We conducted a population-based time series analysis of 2436 478 singleton births in Georgia from 2000–2018, in which individual-level state birth records were aggregated to the county–week level and linked to weekly, county-level TC data from the National Oceanic and Atmospheric Administration and the National Aeronautics and Space Administration. TC exposure metrics included maximum sustained wind speed (>17, 22, 25 m s^−1^), cumulative rainfall (>125, 150, 175 mm), storm proximity (<5, 10, 20 km), and flooding events (yes/no). Generalized linear models with a Poisson distribution were used to estimate relative risks (RRs) and 95% confidence intervals (CIs) for weekly rates of preterm birth (PTB, <37 weeks), low birthweight (LBW, <2500 g), small-for-gestational-age (SGA, <10th percentile), and proportion of male births. Winds >22 m s^−1^ were associated with higher risk of PTB (RR = 1.58 [95% CI: 1.27, 1.96]), LBW (RR = 1.77 [95%CI: 1.40, 2.23]), and SGA (RR = 1.38 [95%CI: 1.12, 1.70]). Rainfall >175 mm was associated with PTB (RR = 1.44 [95%CI: 1.08, 1.93]) and LBW (RR = 2.06 [95%CI: 1.50, 2.83]). Proximity <5 km was associated with PTB (RR = 1.41 [95%CI: 1.04, 1.90]) and LBW (RR = 2.11 [95%CI: 1.38, 3.23]). Flooding was associated with LBW (RR = 1.11 [95%CI: 1.03, 1.21]) and SGA (RR = 1.08 [95%CI: 1.01, 1.16]). Risk estimates were generally higher in rural versus metropolitan counties. Across multiple metrics, TC exposures were linked to increased PTB and fetal growth restriction, with stronger effects in rural counties. These findings bolster information on perinatal risks of TCs and inform more targeted disaster preparedness.

## 1. Background

Tropical cyclones (TCs) (also referred to as hurricanes or typhoons based on region of origin) are rapidly rotating storm systems that originate over tropical or subtropical waters and are characterized by a low-pressure center, strong winds and organized convection [1]. From 1980–2023, TCs caused the most deaths and damage among United States (US) environmental disasters [2, 3]. Modeling studies predict that climate change will magnify the intensity (i.e. wind speed and potential damage) and risks of future TCs [4–7]. The risk of intense TCs was estimated to increase by 1%–5% by 2050 globally [6]. At the same time, extreme wave heights driven by TCs are expected to increase by as much as 1.5 m by 2050 [5]. Moreover, the seasonal peak of category 3+ storms is shifting earlier by about 3–4 d per decade, suggesting that by the 2050s the Atlantic hurricane season could routinely begin in May and overlap more directly with other summer extreme events such as extreme rainfalls [6].

The health impacts of TCs are well-documented for mental health disorders, all-cause mortality, and hospitalizations [8]. However, evidence for birth outcomes is inconclusive [8]. Accumulating evidence suggests an association between TCs and gestation length or preterm birth (PTB) [9–17]. Evidence for growth restriction outcomes, such as low birth weight (LBW) and small-for-gestational-age (SGA), is less consistent. Some studies have suggested that hurricane exposure was associated with LBW [11, 14, 18, 19] or SGA [18, 20], whereas others found no association [9, 13, 21, 22]. Furthermore, male-to-female ratio at birth has been found to decline after natural disasters like earthquakes, due to the increased vulnerability of male fetuses to maternal stress [23]. However, this outcome has rarely been investigated with respect to TCs.

Gaps in our understanding of the adverse birth impacts of TCs may be due to several factors. Inconsistencies in previous studies of TC exposure and birth outcomes may be due to a lack of consistent storm metrics [24]. There is no consensus on the best metric to define TC exposures, and many epidemiological studies use sustained wind speed as an indicator of storm intensity, which could miss other health-relevant aspects of the storms, such as rain and flooding, contributing to incomplete exposure profiles [12]. Many prior studies of TCs employ pre-post storm designs without concurrent control groups. While informative, this approach may be susceptible to temporal confounding from secular trends or co-occurring factors [8, 24]. Time-series designs, by contrast, leverage repeated observations within the same geographic unit, providing effective control of time-invariant confounding. Furthermore, many studies focus on a single storm event, which could be subject to storm-specific confounding and limits generalizability. Few studies have investigated the health burden of TCs in inland areas. Finally, investigation into urban/rural disparities in the impact of TCs on adverse birth outcomes has been limited. Compared to urban populations, rural residents may be more isolated and less resilient to natural disasters [25], experience more significant physical capital loss [26] and slower recovery post-disasters [27]. Pregnant women residing in rural areas have a higher baseline risk of adverse pregnancy outcomes [28], lower educational attainment [29], and lower access to prenatal care or specialists [20, 30]. In addition, rural populations may face lower access to emergency services and greater disruptions to transportation and healthcare infrastructure during disasters. Collectively, these factors potentially widen urban/rural disparities in TC-related adverse birth outcomes.

Many storms track inland across central and northeastern Georgia counties with strong winds and heavy rainfall [31, 32]. However, because Georgia rarely experiences coastal landfalls, the state has received little attention with regard to post-disaster response compared to Florida, Alabama, or the Carolinas, leaving the inland and rural counties in Georgia especially vulnerable [25, 27].

Therefore, our objectives were to evaluate the association between exposure to TCs and several adverse birth outcomes in Georgia from 2000–2018 using a time series study design, to explore different TC-related exposure metrics and cutoffs to present a more comprehensive exposure profile, and to investigate potential urban/rural disparities in the association of TCs and birth outcomes.

## 2. Methods

### 2.1. Study design and study population

We conducted a population-based, time-series study leveraging individual-level birth outcome data and time-varying, county-level TC exposure data in Georgia from 2000–2018. By comparing births within the same county across time, this design inherently controls for many location-based confounders and community characteristics such as socioeconomic conditions, demographics, local climate, and healthcare infrastructure, that could otherwise confound associations. Recognizing that different birth outcomes have distinct critical exposure windows, we focused on short-term exposure metrics that reflect acute conditions surrounding TC events. The study population initially included all registered live births in Georgia during the study period obtained from the Georgia State Office of Vital Records. We then excluded non-singleton births and records with missing or implausible data on date of birth, infant sex, birth weight, gestational age [33] or address information, yielding a final analytic sample of 2436 478 births.

### 2.2. Outcome identification

The following birth outcomes were evaluated with data extracted from the birth records: PTB, LBW, SGA, and the proportion of male births. Gestational age was either clinically estimated or calculated as the complete week difference between child’s date of birth and mother’s last menstrual period. PTB is defined as births delivered prior to 37 complete weeks of reported gestation. LBW is defined as birth weight <2500 grams at delivery. SGA is defined as birth weight <10th percentile for gestational age using a 2017 referent population in the U.S [34]. Infant sex is reported as male or female. We aggregated the number of PTB, LBW, SGA and male births by week and county.

### 2.3. Exposure assessment

Using geocoded residence at birth, we assigned a county to each birth record using ArcGIS Pro 3.3.0. Storm tracks for Atlantic-basin tropical storms between 2000–2018 were obtained from the US National Hurricane Center’s Best Track Atlantic hurricane database (HURDAT2) [35]. Storms that passed within 250 km [12] of at least one county within Georgia were included in the study.

Recognizing that different birth outcomes may be influenced by short-term exposures occurring over distinct etiologic windows, we considered time-varying, county-level TC exposure metrics by lagged exposure weeks. We utilized four distinct storm metrics with varying thresholds to quantify different features of TC-related exposure: maximum sustained wind speed, cumulative rainfall, distance to storm track, and flooding events. We selected cutoffs for maximum sustained wind speed, cumulative rainfall, and distance to storm track based on each variable’s distribution, corresponding to thresholds that classified approximately 30%, 50%, and 70% of counties as exposed. The maximum sustained wind speed at Georgia’s counties’ population mean center (based on the 2010 U.S. Census) [36] was modeled based on the best tracks hurricane tracking data using a model developed by Willoughby *et al* [37]. This approach assigns exposure at the population-weighted centroids of each county, approximating conditions experienced by the majority of residents. A county was considered exposed if the maximum sustained wind speed exceeded the thresholds of 17 m s^−1^, 22 m s^−1^ and 25 m s^−1^. The daily rainfall data in Georgia counties was drawn from hourly, 1/8° gridded data from the National Land Data Assimilation System Phase 2 (NLDAS-2) [38], which is a reanalysis process that integrate multiple weather datasets to create high-quality and comprehensive estimations of weather conditions. The average rainfall in US Eastern counties from 5 d before to 3 d after a storm’s closest approach to the county center were estimated [36]. The cumulative rainfall of 1 d before and 1 d after a storm’s closest approach to Georgia counties was calculated as rainfall exposure. A county was considered exposed if the cumulative rainfall exceeded 125 mm, 150 mm, and 175 mm. Distance to storm track was calculated as the closest distance from the storm track to county’s population mean center. Distances less than 5 km, 10 km and 20 km were used to define exposure to TC using distance to storm track. Flooding events related to TCs were based on data from the National Oceanic and Atmospheric Administration Storm Events Database as reported occurrences of flooding (e.g. ‘Flood,’ ‘Flash Flood’) that occurred in a given county and were temporally and spatially linked to a TC. Flood exposure was defined as any flooding event reported in association with a TC event versus none. The majority of the exposure metric data was accessed through the R package ‘hurricaneexposure’ and ‘hurricaneexposuredata’ developed by Anderson *et al* [36, 39], precipitation data of 2012–2018 was retrieved from the NLDAS-2 Goddard Earth Sciences Data and Information Services Center portal [38]. Correlations between these metrics were examined using Spearman correlation coefficient or Wilcoxon test.

### 2.4. Temperature assessment

We adjusted for temperature in the models because it is associated with storm season and has a well-documented influence on birth outcomes [40, 41]. By controlling for temperature, we reduce the risk that seasonal or storm-related temperature changes could bias our estimates of TC effects. Daily maximum and minimum temperature on a 1 km × 1 km gridded surface were drawn from the Daymet Version 4 dataset [42]. The average daily temperature of the grid cells within Georgia county boundaries was calculated as the daily county mean temperature. The average temperature within weeks was calculated as the mean weekly temperature for each county. Pairwise correlations between weekly average temperature and TC-related exposures were weak (*r* = 0.15 for wind speed, *r* = −0.14 for rainfall, *r* = −0.03 for flooding), indicating minimal collinearity.

### 2.5. Urbanicity/rurality

We used the US Department of Agriculture Rural-Urban Community Areas (RUCAs) codes 2010 version to classify Georgia counties into metropolitan areas and micropolitan, small town and rural areas because RUCA codes capture both population density and commuting flows [43], which is an important dimension of vulnerability during TCs, when disruptions to transportation and workforce mobility can exacerbate risk and impede access to emergency and healthcare services. We used the 10-level primary code in this study [43]: Metropolitan area core: primary flow within an urbanized area (UA); 2. Metropolitan area high commuting: primary flow 30% or more to a UA; 3. Metropolitan area low commuting: primary flow 10% to 30% to a UA; 4. Micropolitan area core: primary flow within an Urban Cluster of 10 000–49 999 (large UC); 5. Micropolitan high commuting: primary flow 30% or more to a large UC; 6. Micropolitan low commuting: primary flow 10% to 30% to a large UC; 7. Small town core: primary flow within an Urban Cluster of 2500–9999 (small UC); 8. Small town high commuting: primary flow 30% or more to a small UC; 9. Small town low commuting: primary flow 10% to 30% to a small UC; 10. Rural areas: primary flow to a tract outside a UA or UC. Because RUCA codes are available at the Census tract level, county-level RUCA code was derived using the mean of population weighted census tract RUCA codes. County-level RUCA code was categorized into two groups: 1–3 (metropolitan areas with low to high commuting flows) and 4–10 (micropolitan, small town and rural areas with varying commuting flows). Sensitivity analysis explored a three-category variable: 1–3 (metropolitan areas with low to high commuting flows), 4–6 (micropolitan areas with low to high commuting flows), 7–10 (small town and rural areas). This classification reflects heightened vulnerability to disrupted transportation, limited healthcare access, and population differences in health status and specialist care [43].

### 2.6. Statistical analysis

Because counts of PTB, LBW, SGA, and male births had a high frequency of zero counts at the county-day level, we aggregated to the county-week level to improve statistical stability while preserving the short-term temporal alignment between TC exposure and birth outcomes. Weeks affected by TCs were defined as weekly maximum exposures exceeding pre-specified thresholds (e.g. the maximum daily sustained wind speed within a given week >17 m s^−1^). County-weeks that were below the exposure thresholds or missing exposure values were considered unexposed: the absence of TC storm data was assumed to indicate the absence of a storm.

We conducted two-stage time series analysis to evaluate the associations between TC-related exposure metrics and weekly rates of adverse birth outcomes. In the first stage, we calculated the county-level relative risks (RRs) and 95% confidence intervals (CIs) comparing the weeks where TC occurred to non-affected weeks within the same county using generalized linear Poisson model with total number of births per week as the offset to account for excess zeros in outcome counts. The model can be written as:

\begin{equation*}\begin{array}{*{20}{l}} {{Y_{ct}}\sim {\mathrm{Poisson}}\left( {{\mu _{ct}}} \right),} \\ {{\mathrm{log}}\left( {{\mu _{ct}}} \right) = {\mathrm{log}}\left( {{N_{ct}}} \right) + {\beta _c}{X_{ct}} + {S_1}\left( {{\mathrm{wee}}{{\mathrm{k}}_t}} \right) + {S_2}\left( {{\mathrm{tem}}{{\mathrm{p}}_{ct}}} \right) + {\gamma _{{\mathrm{yea}}{{\mathrm{r}}_t}}}} \end{array}\end{equation*}

where ${Y_{ct}}$ is the number of outcome events in county c and week t, ${N_{ct}}$ is the total number of births (offset), ${X_{ct}}$ is the TC exposure indicator, ${S_1}\left( \cdot \right)$ is a natural cubic spline of week of year (6° of freedom) to control for seasonality, ${S_2}\left( \cdot \right)$ is a natural cubic spline of weekly mean temperature (4° of freedom) because temperature is associated with storm season and birth outcomes [40, 41], thereby reducing the risk that seasonal or storm-related temperature changes could bias our estimates of TC effects, and ${\gamma _{{\mathrm{yea}}{{\mathrm{r}}_t}}}$ is a categorical indicator for calendar year.

In the second stage, county-specific log-RRs ($\hat \beta {_c}$) were pooled using random-effects meta-analysis:

\begin{equation*}\hat \beta {_c} \sim N\left( {\beta ,{\tau ^2}} \right).\end{equation*}

To obtain overall effect estimates while allowing for between-county heterogeneity in TC impacts. To investigate urban/rural effect differences, we conducted stratified analyses by the binary urban/rural indicator and performed Wald tests to detect significant differences.

We conducted several sensitivity analyses by (1) restricting the LBW outcome models to term births to address potential confounding by gestational age, (2) conducting moving average lag models from lag0–1 to lag0–12 weeks to examine cumulative and delayed effects of exposure, (3) conducting daily-level models (with PTB as outcome) as comparison to the weekly-level models to assess potential exposure misclassification introduced by weekly aggregation, (4) stratifying models using a three‐level urban/rural classification to examine more granular urban/rural levels, and (5) stratifying models by maternal education level (less than high school, high school, college and above) and maternal race/ethnicity (White, Black, Hispanic and other) to examine effect modification by maternal socioeconomic status. All analyses were performed using *R* version 4.4.2 and two-sided *p* < 0 · 05 was used to indicate statistical significance.

This study was approved by the Yale University Institutional Review Board.

## 3. Results

In our study of 2436 478 singleton live births, 242 889 (10.0%) were PTB, 170 074 (7.0%) were LBW, 240 282 (9.9%) were SGA, and 1242 239 (51%) were males (table 1). The prevalence of outcomes by counties is shown in eTable 1. On average, maternal age was 28 ± 6 years. A total of 47% of mothers identified as White, and 33% as Black or African American. Nearly half of mothers (48%) completed college or higher education; 30% had a high school diploma.

**Table 1.** Study population characteristics based on registry-based birth cohort in Georgia (2000–2018).

	All live births (2000–2018), *N* = 2436 478 *N* (%) or Mean ± SD
Preterm birth (<37 wks)	242 889 (10%)
Low birthweight (<2500 g)	170 074 (7.0%)
Small-for-gestational-age (<10th percentile)	240 282 (9.9%)^a^
Male infant	1242 239 (51%)
Term low birthweight (<2500 g)	50 316 (2.3%)^b^
Birthweight (g)	3254 ± 557
Gestational age (wks)	38 ± 2
Maternal age (yrs)	28 ± 6
Maternal race/ethnicity	
White	1140 766 (47%)
Black or African American	793 994 (33%)
Hispanic	349 430 (14%)
Asian	86 459 (3.5%)
Multiracial	42 036 (1.7%)
American Indian or Alaska Native	2924 (0.1%)
Native Hawaiian or Other Pacific Islander	1735 (<0.1%)
Unknown	19 134 (0.8%)
Maternal education	
College or above	1157 491 (48%)
High school	724 684 (30%)
Less than high school	482 690 (20%)
Unknown	71 613 (2.9%)

^a^ Total sample for SGA: *N* = 2434 515.

^b^ Total sample for term low birthweight: *N* = 2193 589.

During the years 2000–2018, 55 TCs passed within 250 km of any Georgia county (efigure 1, eTable 2). Among Georgia’s 159 counties, 109 (69%) experienced peak sustained wind speeds exceeding 17 m s^−1^ during the study period. Stricter wind thresholds were less frequent: 80 counties (50%) had >22 m s^−1^ winds and 49 counties (31%) had >25 m s^−1^ winds. Regarding proximity to storm tracks, 47 county population mean centers (30%) lay within 5 km, 78 counties (49%) within 10 km, and 112 counties (70%) within 20 km. Similarly, cumulative rainfall thresholds of >125 mm occurred in 124 (78%) counties, >150 mm in 84 counties (53%), and >175 mm in 47 counties (30%) (eTable 3). Among all county-weeks, 94.4% had no TC exposure, 5.3% were associated with a single storm, and 0.3% involved two storms occurring within the same week. TC-related exposures were moderately to strongly correlated with one another: wind speed was positively correlated with rainfall (*r* = 0.46) and inversely correlated with distance to track (*r* = −0.78), while all three were associated with flooding occurrence (all *p* < 0.01) (eTable 4).

### 3.1. PTB rate

**Figure 1.**
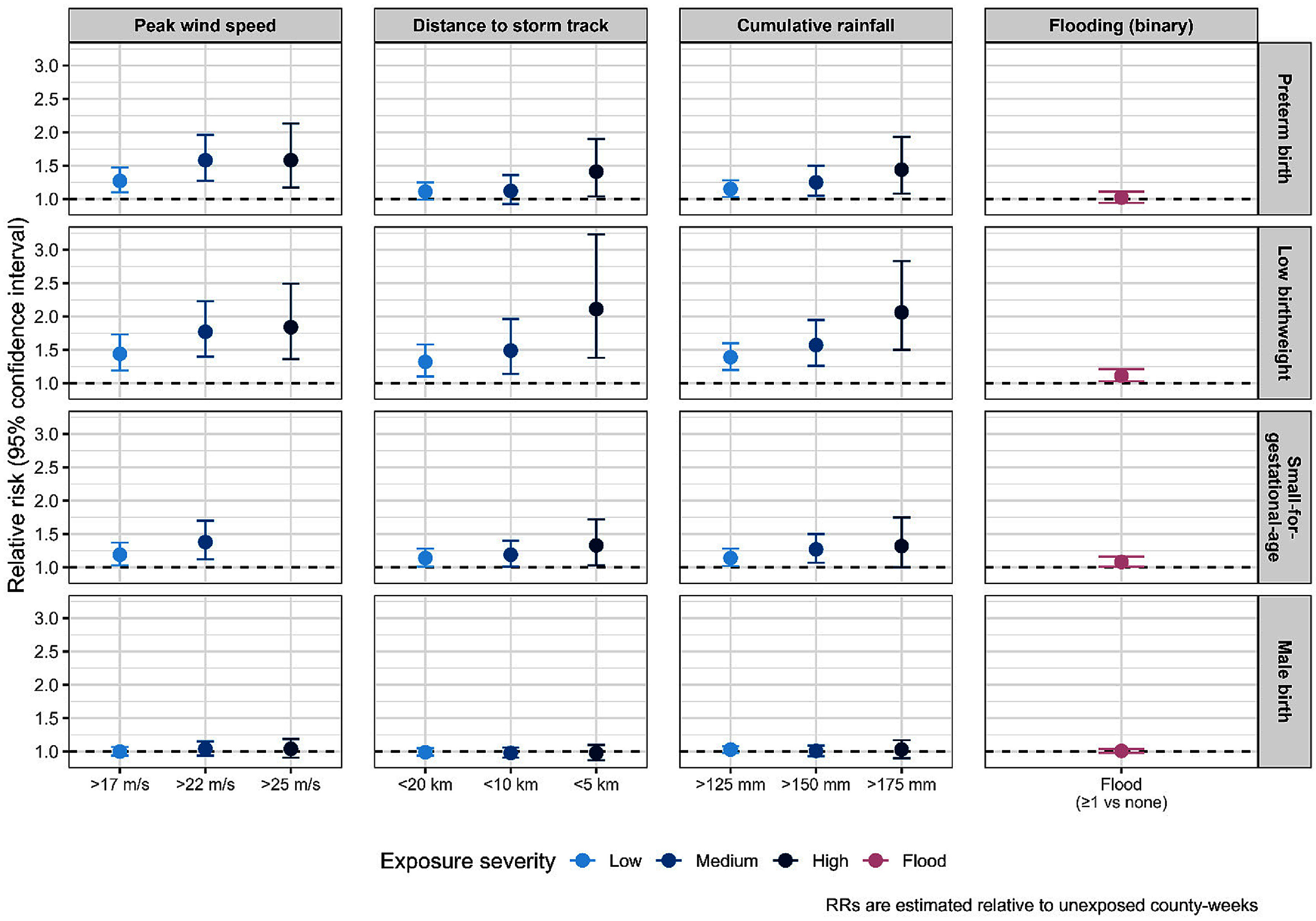
Relative risk and 95% confidence intervals (CI) of tropical cyclone exposure metrics and birth outcomes overall^a^. ^a^the reference group is counties where exposure metric did not meet the threshold during that week.

**Figure 2.**
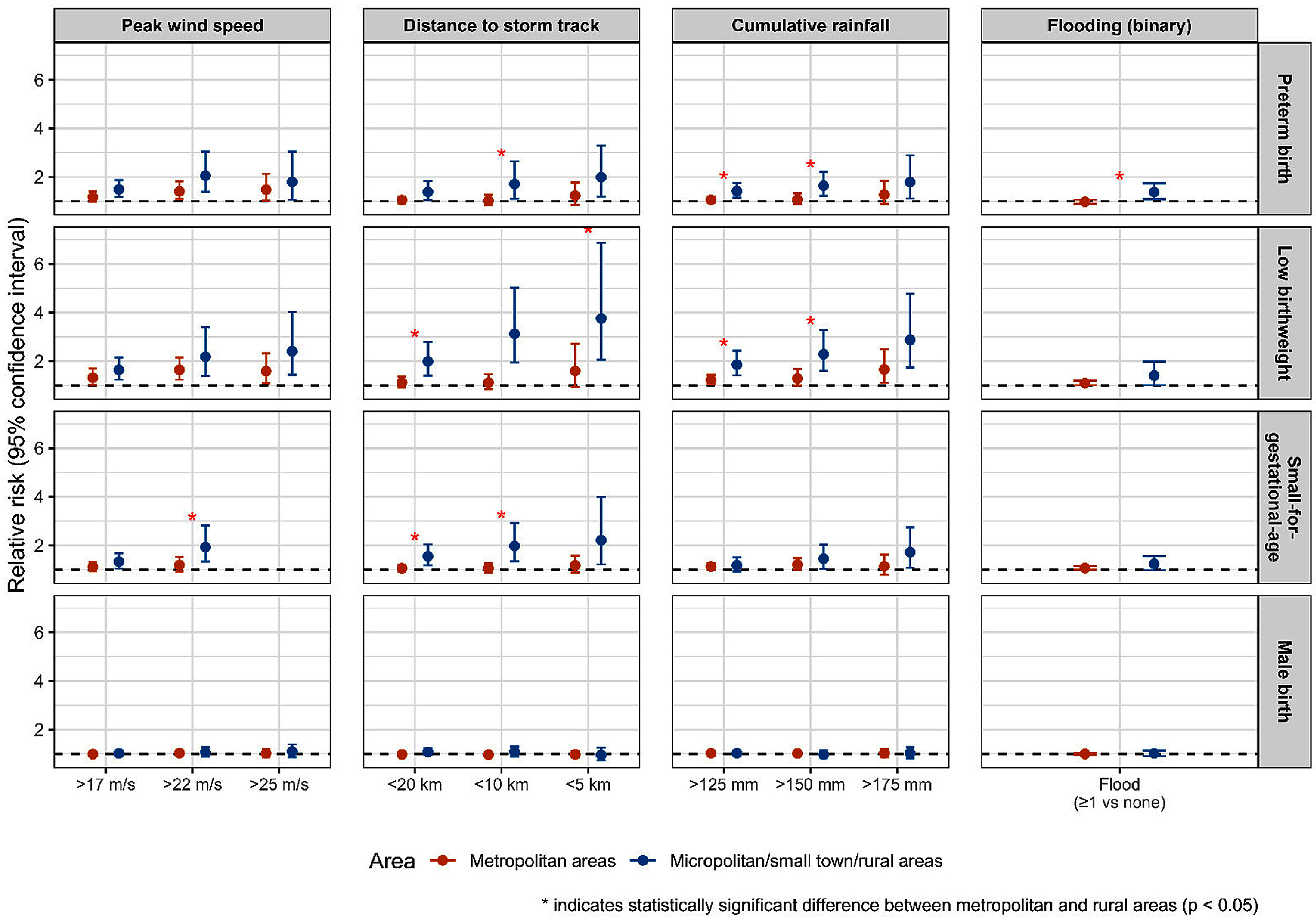
Relative risk and 95%CI of exposure metrics and birth outcomes stratified by rural/urban indicator^a^. ^a^the reference group is counties where exposure metric did not meet the threshold during that week.

All TC-related exposure metrics except flooding events were statistically significantly associated with elevated risk of PTB, with risk estimates increasing in magnitude across higher exposure levels (figure 1, eTable 5). The effect size was largest for peak wind speed: RR = 1.27 [95% CI: 1.10, 1.47] for >17 m s^−1^, RR = 1.58 [95%CI: 1.27, 1.96] for >22 m s^−1^, and RR = 1.58 [95%CI: 1.17, 2.13] for >25 m s^−1^. Flooding was associated with PTB in rural areas (RR = 1.38 [95% CI: 1.09, 1.75]), but not in more metropolitan areas (*P*_wald_ = 0.007) (figure 2).

### 3.2. LBW rate

Higher risks of LBW were observed across TC-related exposure metrics, with larger risk estimates at higher exposure levels. We observed the largest effect size for cumulative rainfall: RR = 1.39 [95% CI: 1.20, 1.60] for >125 mm, RR = 1.57 [95% CI: 1.26, 1.95] for >150 mm, and RR = 2.06 [95% CI: 1.50, 2.83] for >175 mm (figure 1, eTable 5). Risks of LBW were significantly higher in rural areas for distance to track <5 km, <10 km, <20 km, and cumulative rainfall >125 mm and >150 mm (figure 2).

### 3.3. SGA rate

Multiple TC-related exposure metrics were associated with higher risk of SGA (figure 1, eTable 5). The largest effect observed was for peak wind speed >22 m s^−1^ with RR = 1.38 [95%CI: 1.12, 1.70]. Flooding was associated with elevated risk of SGA as well (RR = 1.08 [95%CI: 1.01, 1.16]). Significantly higher risk of SGA was observed in more rural versus more metropolitan areas when considering storm peak wind speed >22 m s^−1^, and proximity <10 km (*P*_wald_ < 0.05) (figure 2).

### 3.4. Male birth rate

Results for male birth rate were null across all TC-related exposure metrics (figures 1 and 2, eTable 5).

**Figure 3.**
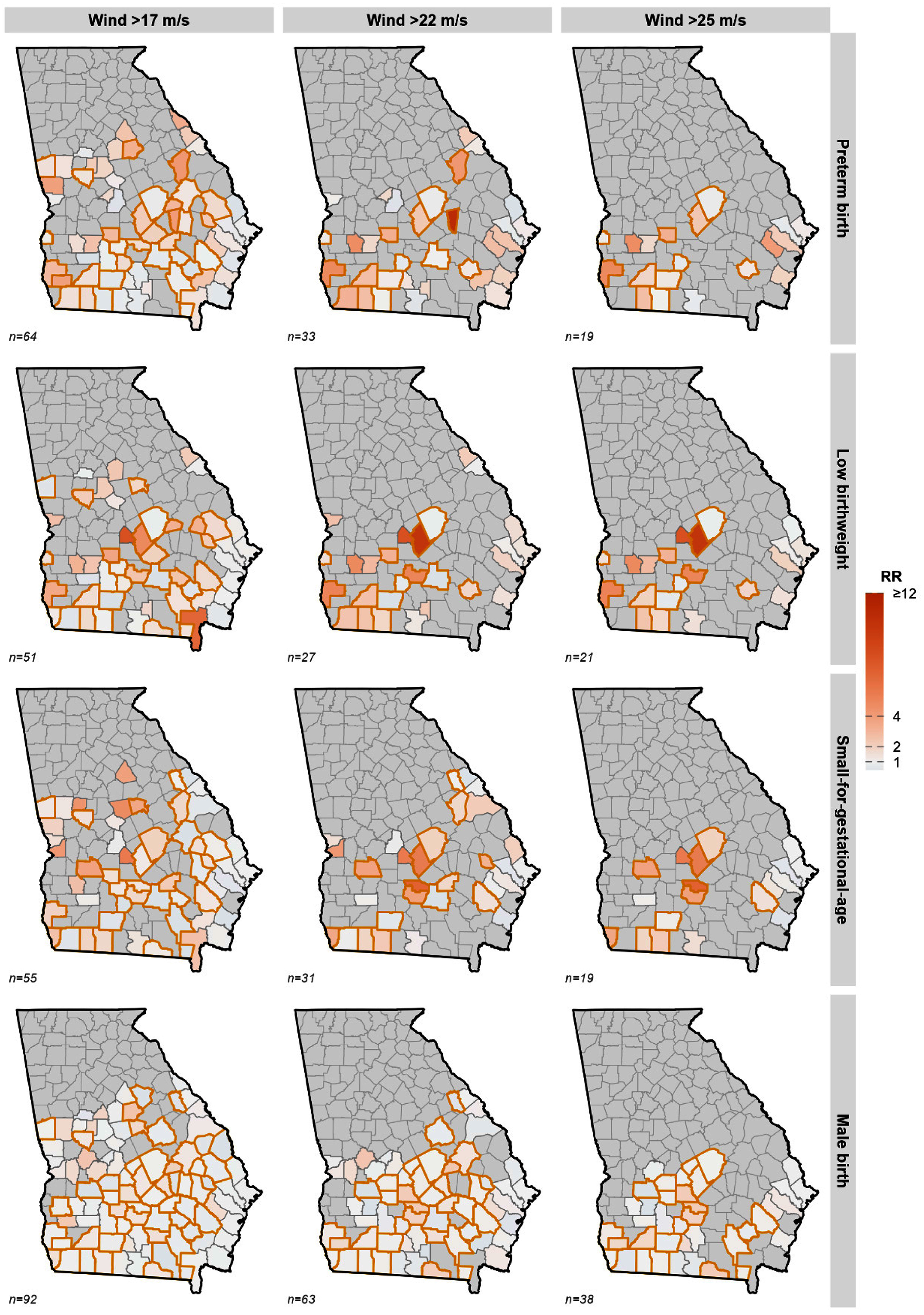
(a) County-specific effect estimates of peak wind exposure and birth outcomes^a,b^. (b) County-specific effect estimates of distance to track exposure and birth outcomes^a,b^. (c) County-specific effect estimates of cumulative rainfall exposure and birth outcomes^a,b^. (d) County-specific effect estimates of flooding exposure and birth outcomes ^a,b^. ^a^ Gray counties were not included in meta-analysis due to no heterogeneity in the exposure. ^b^ Number of exposed counties indicated as *n* = *xx*.

We observed spatial heterogeneity in the county-level risk estimates for PTB, LBW, and SGA across different exposure metrics (figure 3). Elevated risks for wind exposure were concentrated in southern and central counties, while rainfall-related risks were more prominent in northern, southern and central counties. Proximity to storm tracks and flooding showed more spatially dispersed patterns of elevated risk, with flooding affecting the widest geographic areas. Across most TC-related exposure metrics, associations appeared more pronounced in more rural than metropolitan areas, underscoring the disproportionate burden of storm-related reproductive risks in less urbanized regions.

### 3.5. Sensitivity analyses

Analyses for LBW among only term births yielded consistent results (eTable 6). Moving average lag analyses found that week 0 (birth week) estimates captured the bulk of the association and the effects diminished after 4 weeks (efigures 2–5). In sensitivity analyses using daily data, effect estimates were consistently larger (but less precise) than those from weekly models (eTable 7). The analyses using a three versus two category urban/rural indicator were consistent, although some estimates for the most rural stratum were less precise due to lower statistical power (eTable 8). Subgroup analyses by maternal education and race/ethnicity revealed greater magnitude of risk among socially disadvantaged groups (mothers with less than high school education, and non-White mothers) (eTable 9–10).

## 4. Discussion

In this statewide time series analysis of more than 2.4 million individual births in Georgia spanning 19 years, we observed that weekly rate of adverse birth outcomes was associated with TC exposure, indicated by multiple storm metrics. Effect estimates generally increased with higher TC storm intensity. The magnitude of these associations tended to be more pronounced in more rural areas, providing early evidence of urban/rural heterogeneity in these associations. TC-related exposure was not associated with the male birth rate. By assessing multiple, complementary TC exposure metrics, including wind speed, proximity to track, cumulative rainfall and flooding events, our study showed that even less intense storms may be associated with adverse birth outcomes.

Our findings align with several prior investigations that focused on specific, severe hurricanes (e.g. Andrew, Katrina, Charley, Sandy) that reported elevated risks of PTB following storm exposure [9–11, 16, 17] yet diverge from studies showing null results [13, 14, 44, 45], suggesting that heterogeneity in storm intensity, exposure definitions, and analytic design may explain mixed results in the literature. A large‐scale U.S. study across 378 counties (1989–2002) on TCs reported a small but significant increase in PTB risk associated with wind speeds >22 m s^−1^ (RR 1.03; 95% CI 1.00–1.05) and stronger associations for rainfall‐ and distance‐based exposures [12]. Our findings in Georgia show the same direction of association with a larger effect size. Our results represent average effects across a heterogenous set of storm events; across-storm health impacts may vary substantially depending on the storm’s unique characteristics, underlying social factors of the affected population and local infrastructure [46].

Compared to PTB, growth restriction outcomes have been less studied. We found that sustained wind speed, distance to track, and flooding events were positively associated with higher risk of LBW and SGA, while cumulative rainfall was associated with LBW but not SGA. Most prior studies used a pre-post hurricane design to study the effect of a single hurricane event on LBW or SGA and found mixed effects [9, 14, 18, 20, 21, 47]. A couple of studies used longitudinal design linked hurricane exposure during pregnancy with higher risk of LBW [22, 45]. The current study extends existing evidence on hurricane‐related growth restrictions by including less severe storms and offering more granular insights into various storm characteristics. Interestingly, we found that flooding events related to TCs were associated with higher risk of LBW and SGA but not PTB. Although hurricane-related flooding is rarely investigated, a couple of studies have reported higher LBW risk after major flooding events (unrelated to TCs) [48, 49].

Although prior studies of air pollutants (e.g. PM_2.5_) and extreme heat suggested that the most susceptible exposure windows for SGA spans from the preconception period through mid-pregnancy [50, 51], we observed an increased risk of SGA with late pregnancy exposure (week 0). This finding could suggest that week 0 exposure might reflect residual or upstream storm impacts because counties that experienced a TC around delivery are typically the same ones hit during earlier pregnancy as well.

We found no significant associations between male birth rates and TC-related exposures. A study of pregnancies conceived via assisted reproductive technology showed fewer male infants born after Hurricane Katrina [13]. However, other studies of Hurricane Katrina and TCs in Queensland, Australia, observed slight increases in male-to-female ratios [52, 53]. Studying sex ratios at birth is important because a shift can have ramifications in long-term demographic aging [54], reshaping population structure [55] and social stability [56]. However, current study suggests that TCs may not influence sex ratios in the same way as other disasters (e.g. earthquakes) [23].

Spatial analysis revealed that relying on wind speed alone would underestimate the spatial footprint of TC-related risks of adverse birth outcomes. While higher peak sustained winds have identified hotspots of high-risk counties in southern Georgia, both rainfall and flooding exposures generate a far more diffuse pattern of elevated PTB and LBW risks. Different TC exposure metrics reveal distinct spatial patterns and varying county‐level risk hotspots.

To our knowledge, this is among the first studies to examine urban/rural differences in TC impacts on birth outcomes. We used the RUCA codes as a proxy for population density, commuting patterns, and access to healthcare, infrastructure, and other place‐based vulnerabilities in rural settings [43]. These measures capture both chronic structural vulnerabilities, such as reduced access to obstetric and specialty care, lower healthcare resource availability, and longer travel distances for prenatal services [57–62], as well as acute disaster-related constraints, including disruptions to transportation and critical infrastructure, such as electricity and water systems during and after TCs [60, 61]. The stronger associations observed in more rural counties may reflect the interaction between these factors. However, RUCA classifications are a relatively coarse proxy and do not fully capture heterogeneity in healthcare access, infrastructure resilience, or social vulnerability within rural areas [63, 64]. Future studies should incorporate more granular measures of healthcare access and social vulnerability in rural communities to better identify the mechanisms driving these disparities.

There are several potential, related mechanisms by which a storm could influence birth outcomes. The initial, direct impact of storms could produce maternal psychosocial stress and trauma, leading to elevated maternal cortisol and altered maternal-fetal endocrine function. Increased placental corticotropin-releasing hormone and inflammatory cytokines levels from maternal stress could contribute to risk of adverse birth outcomes [65, 66]. Multiple case-crossover and daily time-series analyses have shown that acute environmental stressors can trigger events that are impending. For example, extreme heat and air pollution in the few days before delivery increases the probability of PTB [67–69]. The secondary impact of storms, such as heavy rainfall and flooding, could contribute to power outages, environmental exposures (molds, pollutants, pathogens), and disruption to healthcare services, which can have short-term and long-term effects on preterm delivery and impaired fetal growth [70]. Yet the operational TC risk classification (the Saffir–Simpson scale [1]) relies solely on peak sustained wind speed and thus overlooks other critical drivers of adverse health impacts. Alternative composite metrics that include multifaceted storm hazards could provide a more complete picture of TC risk.

We considered multiple dimensions and features of TCs in this analysis; however, additional refinements could improve their informative value. For example, rainfall exposure was defined using a consistent, fixed window around the estimated closest approach of each storm. While this allowed for a standardized comparison across a large number of events in a time-series framework, it may not fully capture variability in storm-specific rainfall duration or intensity and may include background precipitation not directly attributable to the TC. In addition, we did not explicitly account for air pollution in our models. TCs can significantly alter PM_2.5_ levels through wet scavenging and storm ventilation, which typically lower concentrations [71], or through post-storm recovery activities like debris burning, which can cause localized increases [72, 73]. Future studies could incorporate high-resolution air quality data to attempt to disentangle these joint environmental effects.

Strengths of the study include the use of multiple, complementary TC exposure metrics across varying intensity thresholds, the analysis of a large population-based birth cohort in a comparatively understudied state, and the explicit evaluation of urban–rural heterogeneity in TC-related reproductive risks. This study has some important limitations. First, exposure misclassification is possible due to weekly-aggregated birth data that may not precisely align with storm timing: results from daily-level sensitivity analyses suggest this would bias towards the null. Second, declines in birth rates, migration, or pregnancy loss following major storms could affect registry data [44]. However, the inclusion of less severe TCs likely reduced the impact of evacuation-related bias. Third, the risk of type I error as we examined several birth outcomes across multiple TC-related exposure metrics with different cutoffs; nevertheless, consistency of associations across exposure dimensions and outcomes supports the robustness of the observed patterns. Fourth, subgroup analyses by rurality, educational attainment, and race/ethnicity were exploratory in nature, so these findings should be interpreted with caution. Fifth, using 2010 RUCA codes may introduce misclassification, though binary classifications were highly consistent from 2000–2020 (91%). Lastly, regional variation in infrastructure, disaster preparedness, and response may limit generalizability to other areas.

## 5. Conclusion

By linking temporally resolved county‐level TC exposure metrics, including wind speed, proximity to storm track, cumulative rainfall and flooding events, to over 2.4 million individual birth records, we provide evidence that short-term exposure to severe storms might trigger preterm delivery and fetus growth restrictions. Moreover, pregnant women residing in rural counties face higher susceptibility to these adverse outcomes than those in more metropolitan areas. Our findings have important implications for public health preparedness as climate change intensifies the frequency and severity of TCs [4–7].

## Data Availability

The data cannot be made publicly available upon publication because they are owned by a third party and the terms of use prevent public distribution. The data that support the findings of this study are available upon reasonable request from the authors. Tropical Cyclone Supplement available at https://doi.org/10.1088/2515-7620/ae718b/data1.
